# Recent Progress in Nanotechnology-Based Approaches for Food Monitoring

**DOI:** 10.3390/nano12234116

**Published:** 2022-11-22

**Authors:** Nguyen Nhat Nam, Hoang Dang Khoa Do, Kieu The Loan Trinh, Nae Yoon Lee

**Affiliations:** 1 Biotechnology Center, School of Agriculture and Aquaculture, Tra Vinh University, Tra Vinh City 87000, Vietnam; 2 NTT Hi-Tech Institute, Nguyen Tat Thanh University, Ward 13, District 04, Ho Chi Minh City 70000, Vietnam; 3 Department of Industrial Environmental Engineering, Gachon University, 1342 Seongnam-daero, Sujeong-gu, Seongnam-si 13120, Gyeonggi-do, Republic of Korea; 4 Department of BioNano Technology, Gachon University, 1342 Seongnam-daero, Sujeong-gu, Seongnam-si 13120, Gyeonggi-do, Republic of Korea

**Keywords:** foodborne, food monitoring, nanotechnology, nanomaterials

## Abstract

Throughout the food supply chain, including production, storage, and distribution, food can be contaminated by harmful chemicals and microorganisms, resulting in a severe threat to human health. In recent years, the rapid advancement and development of nanotechnology proposed revolutionary solutions to solve several problems in scientific and industrial areas, including food monitoring. Nanotechnology can be incorporated into chemical and biological sensors to improve analytical performance, such as response time, sensitivity, selectivity, reliability, and accuracy. Based on the characteristics of the contaminants and the detection methods, nanotechnology can be applied in different ways in order to improve conventional techniques. Nanomaterials such as nanoparticles, nanorods, nanosheets, nanocomposites, nanotubes, and nanowires provide various functions for the immobilization and labeling of contaminants in electrochemical and optical detection. This review summarizes the recent advances in nanotechnology for detecting chemical and biological contaminations in the food supply chain.

## 1. Introduction

Since the description of microorganisms was reported by Robert Hooke and Antoni van Leeuwenhoek in the period 1665–1683, scientists studied continuously and successfully explored the tiny world of nature [1]. Consequently, taxonomy systems of microorganisms, such as bacteria and protozoa, were constructed [2]. Additionally, scientists discovered features of extremely small materials and constructed protocols for making nanoscale particles [3]. Increasing knowledge of nanoscale objects led to the formation of “nanotechnology”, first mentioned by Taniguchi in 1974 [4]. Nanotechnology enabled the unique quantum and surface phenomena of the materials. For example, the element carbon can be found naturally in graphite and diamond, of which carbon arrangement exhibits softness and hardness properties, respectively. However, one layer of carbon, called graphene, shows powerful features, such as being harder than diamond, lighter than aluminum, and tougher than steel [5], which enabled applications of graphene in the coating, electronics, sensors, biotechnology, and so on. In addition to graphene, various nanomaterial types were discovered and screened, such as silver, copper, gold, iron, cobalt oxide, and titanium dioxide that boosted development of nanotechnology [6,7,8]. Additionally, different structures of nanomaterials were developed, such as fullerene, nanofibers, nanotubes, nanowires, perovskites, and polymers [9]. In addition to the contribution to development of nanomaterials, nanotechnology-related methods enable detection of harmful tiny particles, such as microplastics, which are found in many objects on Earth [10].

The diversity of types and structures of nanomaterials triggered various applications in many fields [11,12]. For example, in the healthcare field, nanotechnology resulted in the formation of nanomedicine, which applied nanomaterials for diagnostics, medical imaging, nanotherapeutics, vaccines, and regenerative medicine [13]. Additionally, scientists thought about nanobots, which are molecules for fixing the errors in a patient’s body. However, more advanced technologies should be explored to make the nanobots come true [14]. For the environment, nanotechnology contributed significantly to remediation (i.e., removing radioactive ions), water purification, oil spill cleanup, and artificial photosynthesis [15]. For energy sustainability, the development of nanotechnology also helps to resolve the problem of energy conversion, distribution, storage, and usage [16].

**Figure 1:**
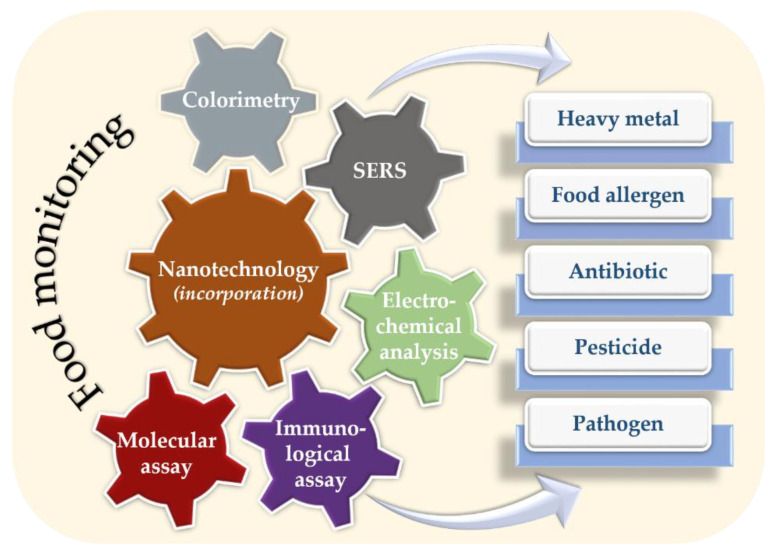
Application of nanotechnology for the monitoring of food contamination.

In the food industry, nanotechnology improved the quality of the products from raw materials to processed items [17,18,19]. For food monitoring, various nano-based techniques were developed, such as molecular assay, immunological assay, electrochemical analysis, surface-enhanced Raman scattering (SERS), and colorimetry (Figure 1). These techniques enabled the detection of heavy metals, pathogens, pesticides, food allergens, and antibiotics during food processing as well as commercial products. Previously, applications of nanotechnology in the food industry were reviewed and discussed [20,21,22,23,24,25]. However, those reviews focused on some aspects of the food industry such as evaluating freshness, packing, and assessments of pathogens. To provide a comprehensive and up-to-date overview of the applications of nanotechnology in the food industry, this review describes factors that affect the food quality and health of consumers, including biotic and abiotic elements. Additionally, it summarizes recent applications of nanotechnology to control and monitor food quality, of which different mechanisms are described.

## 2. Types of Contamination

### 2.1. Chemical Contaminations

One of the main causes of contamination in food is the presence of undesirable chemical compounds or the presence of chemical hazards with a higher concentration than the amount that is considered safe. Massive amounts of chemical hazards in sewage and wastewater can contaminate soil and water, which then enter the food chain through the metabolisms of plants, posing a severe threat to not only humans, but also the entire ecosystem. Due to the fact that humans always stay at the bottom of the food chain, we would accumulate more chemical hazards since the concentration of chemical hazards rises along the food chain [17,26,27,28]. Chemical hazards can also accumulate in food or beverages through the long chain of the production where all stages, such as processing, packaging, storage, and transportation, can become potential sources of chemical contaminants [29,30,31]. Chemical contaminants such as heavy metals, antibiotics, and halogenated compounds can enter the human body and cause severe health problems to humans through foods or beverage consumption. During food processing, undesirable compounds can be produced through chemical reactions or thermal conditions of cleaning, heating, roasting, hydrolysis, or fermentation [32]. Toxic compounds such as polycyclic aromatic hydrocarbons, furanes, nitrosamines, chloropropanols, or acrylamide can be formed during food processing [33]. The use of unapproved adulterants and food additives can become one of the risks to human health [34]. A number of additives, such as antioxidants, plasticizers, stabilizers, and slipping agents, are usually used in the packaging processes, which can be harmful the human health. Those harmful compounds can be transferred to foods through direct or indirect contact between foods and packaging materials. As an obvious example, foods packed in metallic cans can be contaminated by metallic ions due to the corrosion of the cans, in which harmful metal ions are released and migrate to foods [34,35,36,37,38,39,40]. The storage and transportation of foods are other potential stages leading to food contamination. Sunlight is a leading cause of the fast deterioration of foods and packaging materials, which transform safe compounds into unsafe compounds.

Heavy metals can be considered as one of the most dangerous and common chemical contaminants that can be found in foods and beverages. Heavy metals are defined as metallic elements that have a high molecular weight and a higher atomic density than that of water by at least five times [41,42,43,44,45]. Some heavy metals are essential elements for living systems, including humans, as long as they are present in small quantities. Heavy metals such as iron (Fe), copper (Cu), cobalt (Co), zinc (Zn), and manganese (Mn) are required for the growth of living organisms. However, the excessive level of heavy metals in the human body can cause severe health problems and even deaths [46,47,48,49]. Table 1 represents the reference values of heavy metals reported by the Environmental Protection Agency (EPA), Food and Drug Administration (FDA), and Agency for Toxic Substances and Disease Registry (ATSDR).

In the food industry, antibiotics are used as an efficient solution for killing or preventing the contamination of harmful microorganisms. Antibiotics are naturally formed by living organisms or synthesized artificially in the laboratory that have a negative impact on the growth of undesirable microorganisms in the livestock industry [57]. The antibiotics with unapproved quantities presented in animal feed can lead to antibiotic residues in food products such as beef, meat, milk, egg, and fish [58,59,60]. The excessive amount of antibiotics can cause dramatic side effects on human health, such as allergy by penicillin; nephropathy and mutagenicity by gentamicin; carcinogenicity by sulphamethazine, oxytetracycline, and furazolidone; and reproductive disorders by chloramphenicol [61,62,63,64].

### 2.2. Biological Contaminations

Biological contamination occurs when foods are invaded by harmful living organisms or the toxins produced by toxigenic pathogens during any stage of food and beverage production. Unlike chemical contaminations, biological contaminations face the risk of an increase in the number of microorganisms and the rapid spread of infectious pathogens since microorganisms can rapidly reproduce and transmit. There are six types of microorganisms that can cause foodborne diseases: bacteria, fungi, viruses, protozoa, parasites, and prions. Major bacterial contaminations in foods and beverages are caused by the invasion of *Escherichia coli*, *Bacillus cereus*, *Clostridium perfringens*, *Campylobacter*, *Salmonella*, *Staphylococcus aureus*, and various types of vibrios, such as *Vibrio parahaemolyticus* and *Vibrio cholera* [65,66,67,68,69,70]. Dangerously, some bacteria can form biofilms, which are the complex ecosystem of one or more bacterial species immersed in an extracellular matrix, such as polysaccharides, proteins, or exogenous DNA. The biofilms enhance the ability of bacterial pathogens to attach to biological structures (fruits, meat, fish, vegetables, etc.) or to hard surfaces (food industry equipment, dispensing and storage surfaces, etc.). Biofilms also enhance the resistance of bacterial pathogens to the disinfection agents in food processing, such as antibiotics, sanitizers, heating, etc. [70,71,72,73,74,75]. Another problem of bacterial contamination is the number of new antibiotic-resistant bacterial strains rapidly increases and their worldwide spread due to the misuse or overuse of antibiotics. Antibiotic-resistant bacteria contain specific genes that allow bacteria to transform their biological structure, leading to an increase in the survivability of bacterial pathogens under the bactericidal effects of antibiotics [76]. The ability to form biofilms is one of the key factors that decrease the permeability of antibiotics into bacterial pathogens, resulting in the high survivability of the pathogens. Some antibiotic-resistant bacterial strains, such as *Klebsiella pneumonia*, *Pseudomonas aeruginosa*, *Acinetobacter baumannii*, *Enterococcus faecium*, and *Enterococcus faecalis*, were reported as multidrug-resistant bacteria. Recently, as Centers for Disease Control (CDC) reported in 2013 [77], antibiotic-resistant foodborne bacteria, such as *Campylobacter*, *Salmonella typhi*, non-typhoidal *Salmonella*, and *Shigella,* are regarded as dangerous threats to human health. In the United States, antibiotic-resistant foodborne pathogens contribute to more than 2 million infectious cases annually that cannot be treated with typical antibiotics, resulting in about 23,000 deaths [78,79].

Unlike bacteria, most viruses are host-specific and rarely cross the species barrier. Therefore, foodborne diseases caused by viral contamination are mostly caused by foodstuffs contaminated by infected food handlers. However, due to the fact that viruses can rapidly mutate, there is an increasing concern about foodborne outbreaks involving the transmission of viruses from livestock to humans [80]. Hepatitis A virus, enterovirus, human rotavirus, human norovirus, human adenovirus, sapovirus, and parvovirus are some of the most frequent causes of foodborne viral infections [81].

## 3. Nanotechnology

Nanotechnology refers to any fields of science and engineering that deal with dimensions in the nanometer scale (1 to 100 nanometer) involving the manipulation of individual atoms and molecules for the construction of materials, structures, devices, and systems [3,82,83]. Materials at the nanoscale have unique features regarding chemical, physical, and biological properties that differ from their features on larger scales. Interestingly, a large number of new materials were introduced, which provided radically different properties through functioning at nanometer dimensions, where new phenomena are associated with quantum effects and the large surface area-to-volume ratios that cannot be seen in the larger dimensions [84,85]. Properties such as fluorescence, melting point, electrical conductivity, chemical reactivity, and magnetic permeability of the material at nanometer sizes are different from the material at larger sizes. One of the fascinating results of the quantum effects of nanoscale materials is the concept of tunable properties. For example, normal-scale gold exhibits yellow color, while nanoscale gold can appear red, purple, or blue, depending on the size of the gold particles [86]. The electronic properties are significantly changed at the nanoscale level as compared to bulk materials. As a typical example, boron (B) in the bulk form does not have metallic properties, whereas a two-dimensional (2D) network of boron, also known as borophene, is an excellent 2D metal [87]. A non-magnetic element can exhibit magnetic properties at the nanoscale level. For example, a cluster of 13 platinum atoms was confirmed to have extraordinary magnetic polarization, whereas magnetic properties are absent in platinum with the bulk form [88,89]. The catalytic properties of dispersed metal particles with nanometer dimensions pose a significant enhancement as compared to normal-size metal particles [90,91,92,93]. Nanomaterials with these unique features were used to address several problems in many scientific areas and industry, including the food industry. Along with these advanced features, nanotechnology became one of the most promising technologies in the 21st century [94,95,96,97,98,99,100].

## 4. Nanotechnology in Food Monitoring

### 4.1. Detection of Chemical Contaminations

#### 4.1.1. Nanotechnology Incorporated in Colorimetric Analysis

The colorimetric analysis is a technique used to determine the presence of an analyst including heavy metals based on the color change of a dye. Colorimetric analysis usually combines with nanotechnology in order to detect and quantify chemical contaminations in the food industry. As one of the most effective nanotechnologies, metal nanoparticles were usually employed for detecting heavy metals. This technique relies on the color change depending on the size of metal nanoparticles. For example, monodispersed gold nanoparticles (AuNPs) with a diameter of less than 30 nm in an aqueous solution render red color [101]. The presence of heavy metals can accelerate the aggregation of AuNPs to stimulate a red shift in the localized surface plasmon resonance band, resulting in the color change of the aqueous solution [102,103]. Apart from AuNPs, silver nanoparticles (AgNPs) and copper nanoparticles (CuNPs) are widely used in colorimetric detection because they also have plasmonic properties as AuNPs [104,105]. In solution, unaggregated AgNPs with sizes smaller than 30 nm and CuNPs with sizes ranging from 10 to 20 nm have yellow and red color, respectively [106,107]. The presence of heavy metals in foods can be detected through a color shift of metal nanoparticles responding to the aggregation level of metal nanoparticles. A metal nanoparticle can be synthesized through chemical or physical techniques, such as chemical reduction [108], biosynthesis [109], vacuum vapour deposition [110], the electrochemical method [111,112], and solvothermal [113]. Based on the fact that metal nanoparticles tend to aggregate themselves, continuously grow into larger clusters, and finally become precipitated, stabilization is a critical step for metal nanoparticle synthesis. Some stabilizing agents, such as polyvinyl alcohol (PVA), polyvinyl chloride (PVC), cetyltrimethylammonium bromide (CTAB), and polyvinyl pyrrolidone (PVP), are commonly used to produce stable metal nanoparticles with homogenous dispersity [114,115].

**Figure 2:**
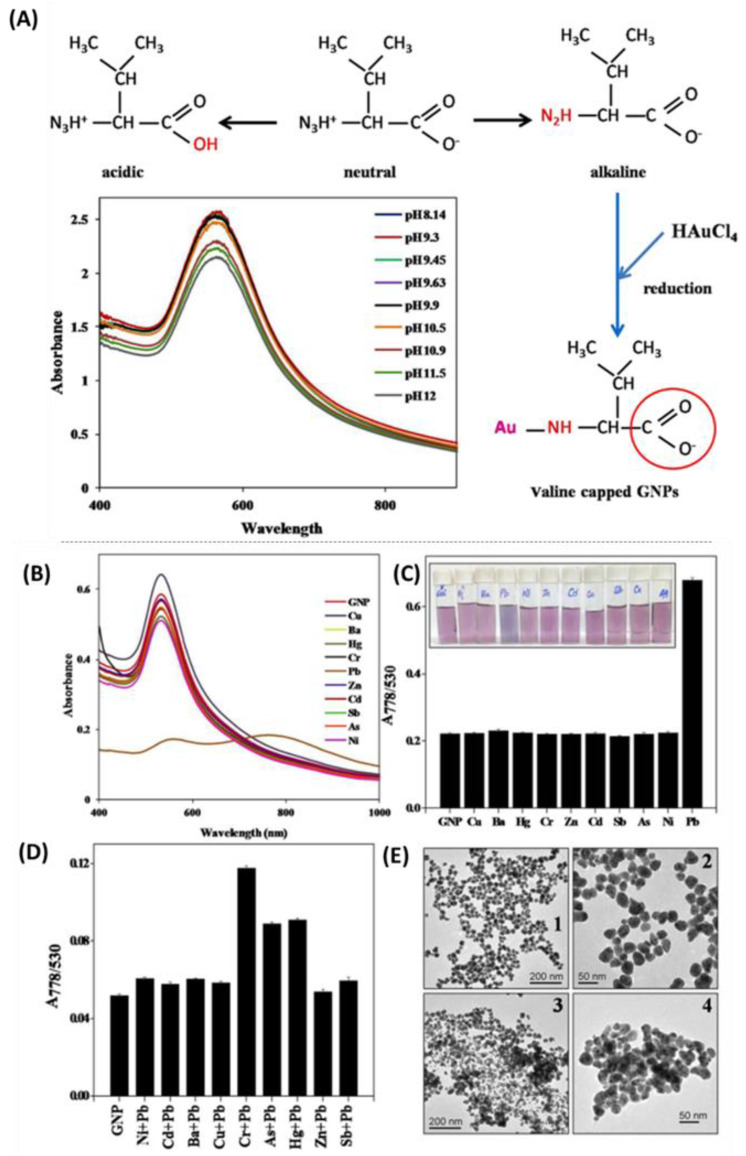
(**A**) Schematic illustration of the mechanism of GNP synthesis and stabilization by valine. Inset shows the effect of high alkalinity of valine capped GNPs. (**B**) Absorption spectra depicting the selectivity of valine-GNPs for Pb^2+^ ions. (**C**) Quantitative analysis of selectivity of valine-GNPs. (**D**) Graph showing the absorption ratio obtained by treating valine-GNP with a commixture of 100 ppm of Pb^2+^ ion and 100 ppm of respective metal ion. (**E**) TEM images of valine-GNPs (**1**,**2**) before treatment with Pb^2+^ ions (**3**,**4**) after treatment with Pd^2+^ ions. Reprinted with permission [116], copyright 2017, Nature Portfolio.

For Pb^2+^ detection, AuNPs is coated with valine, which not only works as a reducing agent and stabilizing agent, but also works as a ligand for the attachment of Pb^2+^ (Figure 2) [116]. In this study, among various tested metal ions (Cu^2+^, Ba^2+^, Hg^2+^, Cr^3+^, Pb^2+^, Zn^2+^, Cd^2+^, Sb^3+^, As^3+^, and Ni^2+^), valine-AuNPs only change from red to blue color in the presence of Pb^2+^, while the presence of all other metal ions retained valine-AuNPs as red in color, and the limit of detection was one ppm for detection of Pb^2+^.

For triple detection of Hg^2+^, Cu^2+^, and Ag^+^, the concept of AuNPs coupled with o-phenylenediamine (OPDA) was introduced by Yang et al. [117]. This technique worked based on the oxidizability of Hg^2+^, Cu^2+^, and Ag^+^ ions toward OPDA and the ability of OPDA to trigger the aggregation AuNPs. Among the tested ions (Fe^3+^, Al^3+^, Cr^3+^, Co^2+^, Ni^2+^, Zn^2+^, Mn^2+^, Mg^2+^, Ca^2+^, Pb^2+^, Cd^2+^, Fe^2+^, K^+^, Na^+^, SO_4_^2−^, CO_3_^2−^, PO_4_^3−^, NO^3−^, NO^2−^, and Cl^−^, Hg^2+^, Cu^2+^, and Ag^+^), only Hg^2+^, Cu^2+^, and Ag^+^ could retain the red color of AuNPs, while the presence of other ions led to the formation of the blue aqueous solution. To distinguish Hg^2+^, Cu^2+^, or Ag^+^ from each other, suitable masking reagents were required, and the limit of detection was 19.5 nM by the naked eye.

**Figure 3:**
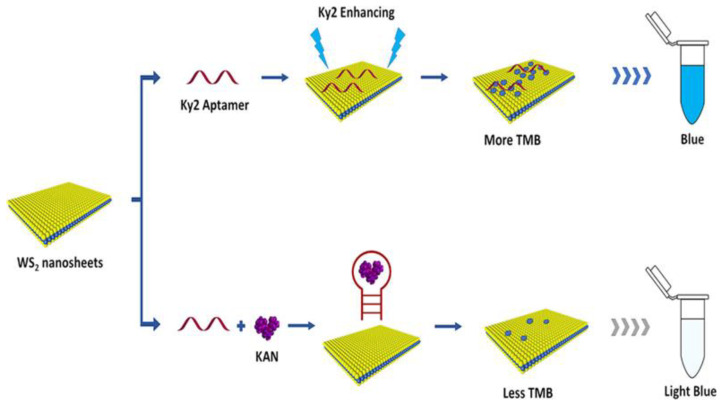
The colorimetric detection of kanamycin residue (Ky2) based on the aptamer-enhanced peroxidase-mimicking activity of the layered WS_2_ nanosheet. Reprinted with permission [118], copyright 2021, American Chemical Society.

Concerning antibiotic detections, kanamycin—an aminoglycoside antibiotic that is widely used in the livestock industry, agriculture, and aquaculture to treat bacterial infection—residue in honey, milk, and pork, Tang et al. [118] developed tungsten disulfide (WS_2_) nanosheets coupled with aptamers and 3,3′,5,5′-tetramethylbenzidine (TMB) (Figure 3). Aptamers (single-stranded RNA or single-stranded DNA) are synthesized oligonucleotides that have high specificity and an affinity toward target molecules including antibiotics [119,120,121]. In the presence of kanamycin, the aptamer can no longer enhance the peroxidase-mimicking activity of WS_2_ nanosheets because of the specific binding of kanamycin to the aptamer, leading to the retention of colorless TMB. The limit detection for kanamycin was as low as 0.06 µM.

Similarly, Ma et al. [122] developed a colorimetric detection of tobramycin in milk and chicken eggs using AuNPs coupled with aptamers.. The presence of tobramycin induced the aggregation of AuNPs, changing color of solution from red to blue, and this approach could detect as low as 23.3 nM from other antibiotics, such as sulfamethoxazole, sulfadimethoxine, sulphachlorpyridazine, streptomycin, and kanamycin.

#### 4.1.2. SERS Analysis

##### Principles of SERS

Surface-enhanced Raman scattering (SERS) is an advanced technology that is extensively used for the ultrasensitive detection of contaminants in the food industry. SERS technology was developed after the discovery of Raman spectroscopy by Raman C.V in 1928. When an incoming excitation light interacts with matter, inelastic scattering of photons is produced in which the wavelength of the scattered photons is distinguished from the incoming light [123,124]. The Raman spectrum provides information about the rotation and vibration of the analyzed molecules, opening a novel fingerprint for molecular recognition. However, the Raman scattering effect is generally 10^10^–10^14^ times lower than infrared and fluorescent signals, making the Raman scattering effect not applicable for the recognition of contaminants at low concentrations [125,126,127]. Scientists discovered that when the analyzed samples are located in close proximity or adsorbed to a nanostructured metal (such as gold and silver), the incident light passing through the sample produces a massive intensification of Raman scattering, also known as SERS [128,129,130]. The controllable number and location of plasmonic nanoparticles on the SERS platform allows the high reproducibility and sensitivity of detection compared to the free-assemble nanoparticle [131,132].

##### Heavy Metals Detection

For heavy metals detection, a simple SERS substrate was simply fabricated by the inkjet printing of AgNPs ink layer (about 400 nm thick) on a silicon wafer [133]. As compared to the bare silicon substrates, the AgNPs-printed SERS substrates could enhance three, four, and five times the Raman scattering for cadmium sulfide (CdS), zinc oxide (ZnO), and mercury sulfide (HgS) detection, respectively. As another example, Barimah et al. [134] synthesized SERS nanomaterial using cuprous oxide (Cu_2_O) nanoparticles and AgNPs for arsenic (As) detection. The SERS sensor proved high selectivity, as it induces a significantly higher SERS intensity at the Raman peak at 811.29 cm^−1^ than other metal ions, such as Cd^2+^, Pb^2+^, Mn^2+^, Ni^2+^, and Hg^2+^, and this sensor could detect as low as 5.61 ppb.

Mercury, which generally is contained in saltwater species, agricultural products, and water, can cause problems in the respiratory system, nervous system, and kidneys [135,136,137]. A SERS model coupled with the colorimetric detection of mercury ions was developed by Song et al. [138]. In this study, Au@AgPt nanoparticles were synthesized to integrate SERS sensors for ultrasensitive detection and naked-eye detection of mercury ions. In particular, SERS coupled with colorimetric detection improves the limitation of SERS analysis, which is dependent on expensive equipment, limiting its applications in low-resource settings. The mercury ion could be detected as low as 0.52 µM by the naked eye, while the lowest concentration of mercury ion that could be detected by SERS signals was 0.28 nm.

He et al. developed a reusable SERS-based sensor for lead detection [139]. In this study, SERS substrate was synthesized by depositing a nanolayer of Ag and Au and a monolayer of graphene on a porous gallium nitride substrate, and the thiolated probe (Cy3-DNAzyme) for hybridization of single-stranded DNA. In the absence of lead, Cy3 labeled DNAzyme hybridized with the single-stranded DNA to form rigid double-stranded DNA, which increased the spatial distance between the Cy3 label and SERS substrate, resulting in the weakness of Raman signals. Meanwhile, the presence of lead could split the complex of single-stranded DNA and Cy3-DNAzyme, in which turn, the Cy3 label close to the SERS substrate resulted in the enhancement of SERS signals. Typically, a Raman vibration of Cy3 has peaks at 1193 cm^−1^, 1391 cm^−1^, 1465 cm^−1^, and 1586 cm^−1^ [140]. The limit of detection is 4.31 pM for lead detection.

##### Detection of Food Allergens

Food allergens are substances that can cause the body’s immune system to react because of an immunological mechanism involving IgE antibodies in the human body. Food allergens are normally not harmful, but they have negative impacts to a susceptible person. Therefore, it is vitally important to determine the food allergens to alert the consumer. The most common food allergens are *β*-conglycinin, agglutinin, Ara h1, lactoferrin, and *β*-lactoglobulin [141,142]. Basically, SERS technology for sensing food allergens relies on the specific interaction between ligands attached to the SERS substrate and the allergens. There are two main types of ligands for capturing food allergens, which are antibodies and aptamers [143].

**Figure 4:**
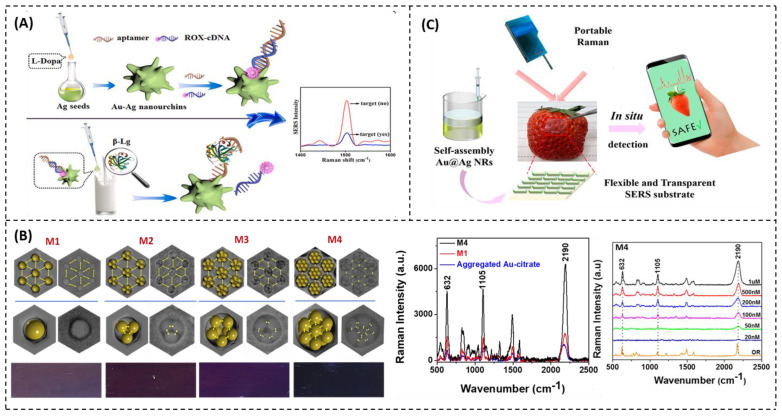
The application of SERS for food monitoring. (**A**) SERS-based aptasensor for β-lactoglobulin (β-Lg) determination using Au-Ag nanourchins as active substrates. Reprinted with permission [148], copyright 2022, Elsevier. (**B**) Controlling numbers of gold nanoparticles in the highly ordered AAO nanopore as SERS substrates with the limit of detection of 20 nM. Reprinted with permission [179], copyright 2019, American Chemical Society. (**C**) In situ detection of pesticide residue by flexible and transparent Au@Ag nanorid array. Reprinted with permission [177], copyright 2022, MDPI.

Typically, antibodies can be deposited on the surface of the nanostructured metal substrate—SERS substrate—through electrostatic forces, biotin–avidin specific adsorption, and covalent immobilizations [144]. An antibody-based SERS sensor was developed for ultrasensitive α-lactalbumin detection in raw cow milk, ultra-heat-treated cow milk, walnut milk drink, and peanut milk drink [145]. The antibody-based SERS sensor could directly detect α-lactalbumin with a limit of detection as low as 0.01 ng/mL. As another example, a gold nanoparticle-based SERS was coupled with immunoassay strip tests, namely SERS-LFA, to detect *β*-conglycinin in soybean [146]. The SERS-LFA assay could detect *β*-conglycinin by both colorimetric detections for naked-eye detection and SERS-based detection for ultrasensitive *β*-conglycinin recognition. The SERS-LFA technique had the ability to accurately quantify the level of *β*-conglycinin from 160 to 100 μg/mL in samples. The limit of detection for *β*-conglycinin was as low as 32 ng/mL. In another work, bimetallic Au-Ag nanourchins were synthesized for *β*-lactoglobulin detection in milk samples (Figure 4A) [147]. The aptamers were hybridized with Raman reporter molecules, namely 6-Carboxyl-X-Rhodamine-labeled complementary DNA (ROX-cDNA). Au-Ag nanourchins massively released Raman signals in the absence of *β*-lactoglobulin because of the close distance between Au-Ag nanourchin and the attached ROX-cDNA on the surface of Au-Ag nanourchins. Meanwhile, the presence of *β*-lactoglobulin significantly decreased Raman signals because *β*-lactoglobulin could break the aptamer-ROX-cDNA complexes, keeping ROX-cDNA away from Au-Ag nanourchins. Au-Ag nanourchins could detect β-lactoglobulin as low as 0.07 ng/mL. Therefore, the nanourchin structures, also known as nanoflowers, provided a significant enhancement of Raman signals due to the sharp tips (which acted as nanoantennas) and the roots of tips (which acted as nanogaps) of the nanourchin structures, resulting in a larger surface roughness and an ultra-high density of hot spots, as compared to sphere nanostructures [148,149].

##### Detection of Antibiotics

The potential of the SERS-based detection of antibiotics in foods was recently addressed. Fang et al. developed an aptamer conformation cooperated enzyme-assisted SERS method for the detection of chloramphenicol (CAP) in milk samples with the limit of detection as low as 15 fM [150]. Jing et al. reported a AgNP-decorated TiO_2_ nanotube array for SERS-based detection of 2-mercapto-5-methyl-1,3,4-thiadiazole (MMT), which is an effective marker for the detection of the degraded antibiotics in milk samples [151]. This SERS-based platform can detect MMT in the concentration range of 0.5−1000 µM, and the limit of detection was confirmed as low as 0.11 µM. The AgNP-decorated TiO_2_ nanotube array coupled with a solid-phase microextraction (SPME) surface served as an all-in-one SERS substrate for both extraction and detection of antibiotics in milk samples. Similarly, Cui et al. developed an SPME-SERS substrate by co-deposition of reduced graphene oxide and silver on silver-copper alloy fibers for sulfadiazine and sulfamethoxazole analysis in tissue mimic. As the reference peaks for sulfadiazine and sulfamethoxazole quantitative analysis, 1149 and 1144 cm^−1^ were chosen, respectively [152]. The sulfa drugs, including sulfamerazine, sulfamethazine, and sulfamethoxazole were also analyzed by SERS technology by Lai et al. [153]. All sulfamerazine, sulfamethazine, and sulfamethoxazole were detected as low as a level of 10 ng/mL (about 10 ppb), which were ten times lower than the acceptable maximum residue limits (MRL) in foods (100 ng/mL or 100 ppb). Alternatively, liquid–liquid extraction (LLE) combined with SERS could serve as an all-in-one platform for the extraction and identification of sulfamethoxazole spikes in biofluids [154]. The LLE-SERS method could detect sulfamethoxazole with the limit of detection of 1.7 µg/mL within 30 min. Tian et al. fabricated bimetallic Au@Ag core-shell nanorods with precise and controllable Ag shell thickness (from 2.1 to 14.1 nm) for the identification of levofloxacin, which belongs to the third-generation fluoroquinolone antibiotic drug [155]. The Au@Ag nanorods, with 7.3 nm Ag shell thickness, provided the strongest SERS signals as compared to other investigated nanostructures. The limit of the detection of levofloxacin was 0.37 ng/L (10^−9^ M). The SERS technique was also used to detect other antibiotics, such as enrofloxacin, ciprofloxacin, and chloramphenicol as low as 20 ppb [156]; moxifloxacin as low as 0.085 µg/mL [157]; benzylpenicillin sodium as low as 10^−7^ mol/L [158]; carbenicillin disodium as low as 0.63 × 10^−8^ mol/L [159]; difloxacin hydrochloride, danofloxacin, and enoxacin as low as 4.36 × 10^−12^, 3.16 × 10^−11^, and 3.15 × 10^−10^ mol/L, respectively [160]; and rhodamine 6G and p-aminothiophenol as low as 5.0 × 10^−11^ and 1.6 × 10^−10^ M, respectively [161].

##### Detection of Pesticides

Pesticide residue is one of the most significant sources of toxicity in water and food. A variety of pesticides are prohibited because they are not biodegradable and impact human health [162]. Chemical pesticides were also identified as causing birth defects in children and cancer [163,164]. The contamination of organochloride pesticides in food products and water are also concerning, due to the danger in long-range transport, bioaccumulation in human and animal tissue, and biomagnification in food chains [165]. The author found that the SERS analysis of organochloride pesticides is limited by their low affinity toward the substrates or poor analytical reproducibility. Recently, the monitoring of acetamiprid, a widely used broad-spectrum and contact insecticide, by SERS substrate, was reported (Figure 4B) [166]. In this work, high-ordered and arranged plasmonic nanoparticles exhibited the limit detection to be ~20 nM, with a relative standard deviation (RSD) of 6.64%. The main characteristic peaks of acetamiprid were obtained at 632, 1105, and 2190 cm^−1^. At present, the development of nanomaterials for SERS substrates were dramatically attracted due to their high advantages of fast and simple techniques for detection of pesticides [167]. Chu et al. describe the use of oyster shells as a green chemical source to prepare calcium oxide nanoparticles, which was then developed as a SERS substrate of silver/polydomanine/calcium-oxide. The limit of detection of methyl parathion, an organio-phosphorus pesticide, is 0.9 nM [168].

Recently, a large number of SERS substrate platforms were developed to monitor several different types of pesticides contained in foods. Deltamethrin, which is a benzim-idazole derivative belonging to the pyrethroid family, is widely used in fruits as an insecticide and fungicide. The detection of deltamethrin was reported to be obtained in strawberries by SERS-based AuNPs enhancement [169], in brew tea by liquid SERS-based Au-Ag colloidal NPs, in wheat by SERS-based Ag@ZnO nanoflowers [170], and in *Corydalis yanhusuo* by SERS-based multi-walled carbon nanotubes [171]. Fipronil, a broad-spectrum insecticide that belongs to the phenylpyrazole family, has hepatoxicity and neurotoxicity in humans [172]. Ly et al. described density functional theory (DFT) and SERS study to detect fipronil by the SERS-based AgNPs platform, which could be used to detect fipronil in eggshells and liquid eggs in the water and soil environment [173]. The core/shell structure of SiO_2_ and AuNPs was developed for the inspection of fipronil in chicken eggs and other foods [174]. Recently, Logan et al. reported a handheld SERS that allows detection limits for three out of four pesticides below the maximum residue limits (MRLs) of 10 ppb in Basmati rice [175]. The multiplexing characteristics of the as-developed handheld-SERS platform assesses in solvent and matrix conditions and has great potential for the rapid on-site detection of pesticide residues in rice and other commodities [175]. The multi-class of four common pesticides of atrazine, simazin, irgarol, and diuron was successfully detected at millimolar concentration by SERS based silver nanospheres and silver nanoprisms [176].

The flexible and transparent SERS platforms, which are composted of Au@Ag nanorod array, were developed for in situ detection of pesticide residues on fruits and vegetables (Figure 4C) [177]. The monitoring of thiram in contaminated strawberries, apples, and mushrooms reached the limit of detection of 2 ng/cm^2^ with high measurement recovery and reproducibility. The portable Raman spectrometer was used for the in situ inspection of the foods. In the same routine, the new type of elastic, flexible, and transparency SERS platform made of poly (ethylene terephthalate) (PET) were integrated with a layer of indium tin oxide (ITO) and AgNPs to detect and identify pesticides on the surface of fruits as trace pesticide analysis. The PET/ITO/Ag SERS platform exhibits the limit of detection of ~2.5 and ~0.012 µg/mL for thiram and carbaryl, respectively, when obtaining on the apple skins [178].

SERS technology can be considered a promising technology for the analysis of toxic elements in foods due to the ability to detect the toxic elements at ultralow concentrations. Interestingly, SERS can combine with colorimetric detection to achieve the naked-eye observation of the results; or combine with extraction methods, such as solid-phase microextraction and liquid–liquid extraction to serve as an all-in-one platform.

#### 4.1.3. Electrochemical Analysis

##### Principles of Electrochemical Analysis

The electrochemical analysis is a collection of analytical techniques that use a measurement of potential, charge, or current to quantify or identify an analyte based on its chemical reactivity. The mechanisms of electrochemical detection can be categorized into two main strategies: non-affinity detection and affinity detection [179]. The non-affinity detection is used for detecting a specific group of analytes, which is electroactive. The electroactive substances can be oxidized or reduced at a certain voltage. Some toxic elements in foods that have strong electroactivity can be directly detected by electrochemical methods. Different from non-affinity detection, affinity-based electrochemical analysis relies on the affinity between the analytes and the receptors, such as aptamers, antibodies, and molecularly imprinted materials [180,181]. The affinity-based electrochemical analysis can achieve detections toward almost all types of toxic elements in foods with high specificity, as compared to non-affinity detections.

Electrochemical analysis, with the assistance of nanomaterials, can massively enhance electrochemical performance. The large surface area-to-volume ratios of nanomaterials allow the electro-catalytic processes and substantially enhance the sensitivity of materials with larger sizes. Therefore, various nanomaterials were developed to amplify electrochemical signals for ultrasensitive detection [182,183]. There are two main strategies for enhancing electrochemical signals using nanomaterials: electrode modifiers and signal tags.

##### Nanomaterials as Electrode Modifiers for Food Analysis

Nanomaterials with excellent conductivity are powerful property that can significantly improve the electrochemical signals by increasing the effective surface area of the electrode and boosting the rate of electron transfer on the electrode surface. Generally, an electrode can incorporate diverse nanomaterials, such as carbon nanotubes, quantum dots, graphene, metal, and oxide nanoparticles to improve analytical performance due to their conductive properties and biocompatibility [184,185,186,187]. The electroanalytical sensors can be modified with single nanomaterials, a binary composite, or triple and multiple nanocomposites [188].

Carbon nanotubes with their unique spatial structure—a hollow tubular one-dimensional nanostructure—became one of the most effective electrode modifiers. Various electrochemical properties of carbon nanotubes, such as electrical conductivity, catalytic activity, stability, and biocompatibility are critical for the detection of toxic compounds in foods using electrochemical methods. Chen et al. developed carbon nanotubes-coated electrodes to significantly increase the surface areas of electrodes for detecting carbofuran, which is a carbamate pesticide [189]. The carbon nanotubes-coated electrodes exhibited excellent sensitivity with the limit of detection of carbofuran as low as 0.1 ppb in an aqueous solution. The electrochemical approach offers a rapid method to analyze the metal ions’ contamination in water, and different nanostructures were designed to modify carbon paste electrodes for the detection of the analytes. A high electrocatalytic activity sensing of Hg^2+^ was obtained in a cupric oxide/polyvinyl alcohol nanocomposite-modified glassy carbon electrode. The limit of detection was 0.42 nM and could be carried out in different water samples [190]. Two types of thoria nanoparticles were applied to carbon paste electrodes for the determination of arsenite and total inorganic arsenic in water with a limit of detection of 0.1 µg/L [191]. As another example, the modified electrodes with carbon nanotubes were also used to detect heavy metals, such as cadmium and lead in herbal food supplements [192]. The limit of detection of cadmium and lead was 1.06 ppb and 0.72 ppb, respectively. Alternatively, ultrasensitive electrochemical microelectrodes modified by carbon nanotubes were used to detect lead with a limit of detection as low as 400 ppt, which is well below the permissible limit for lead (10 ppb) reported by the WHO [193]. The modified electrodes with carbon nanotubes also pose electrochemical performance with great sensitivity for antibiotics detection. The limits of detection of ciprofloxacin, chloramphenicol, and furazolidone were 5 nM [194], 10 µM [195], and 0.03 µM [196], respectively.

As another strategy for electrode modification, quantum dots as zero-dimensional nanomaterials with negligible size and extremely high surface-to-volume ratio are excellent nanomaterials. Quantum dot-based electrodes could be used to enhance the sensitivity of aflatoxin B1—a toxin produced by fungus occurring in food, i.e., grains, peanuts, maize, and groundnuts—detection in maize samples with a detection limit of 0.09 ng/mL [197]. The quantum dots@porous carbon platform for the electrochemical detection of oxytetracycline—an antibiotic—was developed by Lin et al. with the limit of detection of 3.23 × 10^−9^ mol/L [198]. Recently, the electrochemical analysis system-based nanostructure was concerned with the detection of organic molecules in foods. In fact, the voltammetric detection of caffeine was reported to be obtained in pharmacology and beverage by the platform of nano Cobalt (II, III) oxide-modified carbon paste electrode in aqueous and micellar media with a limit of detection of 0.016 µmol/L [199], in tea stuff by Co_3_O_4_/GCE nanofion electrode with a limit of detection of 0.097 µM, and in tea and coffee by Cu-MOF/graphene composite with a limit of detection of 1.38 µM. Typically, the modified electrode exhibited high sensitivity, stability, and reproducibility. The electrochemical manners of caffeine could be characterized by cyclic voltammetry (CV), electrochemical impedance spectroscopy (EIS), and chronoamperometry (CA). In foods, some types of food additives may be harmful to human health, such as preservative and colorant ingredients. For example, sodium nitrite, the most common food preservative, is associated with an increased risk of stomach cancer, bladder cancer, and thyroid cancer [200]. A modified electrode of iron (III) tetra-(N-methyl-4-pyridyl)-porphyrin and copper tetrasulfonated phthalocyanine was designed to monitor nitrile compounds in foods with a limit of detection of 0.1 µmol/L [201]. A novel biosensor of ZnO nanoparticles and poly (*p*-aminobenzensulfonic acid) was described in the detection of tartrazine, a colorant adulteration in foods, with a limit detection of 80 nM [202].

##### Signal Tags

The electrochemical signals can be improved through the combination of signal tags. Signal tags are the labeled elements attached to the surface of the electrode in order to produce electrochemical signals for analyzing the analytes. Nanomaterials with excellent electrochemical activity serve as promising materials for generating catalytic signals through catalyzing biochemical reactions [117,203]. Wei et al. synthesized thymine-functionalized silver nanoparticles (Ag-T) as the signal tags for mercury detection [204]. In general, the presence of mercury induced the aggregation of Ag-T nanoparticles, resulting in the enhancement of electrochemical signals. Meanwhile, the absence of mercury retained the dispersion of Ag-T, resulting in the decrease in electrochemical signals. The detection limit of this mercury electrochemical sensor was 5 pM.

### 4.2. Detection of Biological Contaminations

For the detection of biological contaminations, a wide range of analytical techniques were developed. Conventional techniques for determining biological contaminants relate to culture-based methods, immunological assays, and molecular assays. Although conventional techniques can effectively detect biological contaminants in foods with high accuracy, sensitivity, and selectivity, these techniques are time-consuming and require expensive equipment. Along with significant advances in nanotechnology, the limitations of current techniques for biological contaminant detection in foods can be improved [205,206,207,208].

#### 4.2.1. Nanotechnology Incorporated in Immunological Assays

The working principles of immunological assays rely on the specific interaction of antibodies to corresponding antigens, including lipopolysaccharides, proteins, and other molecules on the surface of biological agents [209,210]. Among various immunological assays, lateral flow immunoassay is an outstanding technique because it allows rapid on-site detection of biological contaminants in foods. Generally, the lateral flow immunoassay involves a paper strip, which is made up of four pads that are arranged orderly as follows: sample pad, conjugate pad, nitrocellulose membrane, and wicking pad. After sample fluid is loaded on the sample pad, it will migrate along the four pads of the lateral flow strip by capillary action. At the conjugate pad, the analytes are labeled by color particles, then flow to the lines in the nitrocellulose membrane and are captured by the attached antibodies or antigens on the lines. The immobilization of the labeled analytes by antibodies or antigens leads to color change, which can be observed by the naked eye [211,212,213].

Recently, nanobodies garnered considerable attention from scientists due to their potential ability to improve the specificity of lateral flow immunoassays. Nanobodies consist of only two heavy chains with a single variable domain as the position for antigen binding. Nanobodies present unique properties, such as nanoscale sizes, the ability to recognize difficult-to-access antigen epitopes, and high specificity, as well as have a high affinity for only one cognate target [214,215]. A nanobody-based immunological biosensor for colorimetric and photothermal dual-mode detection of *Salmonella typhimurium* spikes in honey, juice, and chocolate samples was developed by Zhang et al. This nanobody-based immunological sensor has a sensitivity of 10^4^ CFU/mL in colorimetric mode and 10^3^ CFU/mL in photothermal mode [216]. He et al. developed an immunoassay with nanobody Nb 13 for *S. enteritidis* in milk [217]. The assay exhibited a limit detection of 1.4 × 10^5^ CFU/mL of *Salmonella enteritidis* in milk after 10 h of enrichment.

Apart from nanobodies, nanomaterials can serve as a good strategy for target recognition and detection. Fu et al. synthesized MnO_2_ nanoparticles coated with polyclonal IgG antibodies for the recognition of *Vibrio parahaemolyticus* and also synthesized AuNP for colorimetric detection [218]. This nanoparticle-assisted immunoassay possessed high specificity with the limit of detection of 10 CFU/mL. Ilhan et al. reported a typical example of AuNP as labels in lateral flow immunoassay for Salmonella enteritidis detection in chicken and egg samples [219]. Xia et al. developed gold magnetic bifunctional nanobeads (GMBN) for an immunochromatographic test strip for efficient detection of *Salmonella choleraesuis* in milk [220]. The sensitivity of the strip for *S. choleraesuis* detection was 5 × 10^5^ CFU/mL. Rodríguez-Lorenzo et al. synthesized gold nanostars for a SERS-based method of ultrasensitive detection of *Listeria monocytogenes* [221]. Gold nanostars were coated with an antibody-specific monoclonal for SERS-based detection of *L. monocytogenes*. This immunoassay-based SERS technique could discriminate *L. monocytogenes* and *Listeria innocua* in just 100 s.

**Figure 5:**
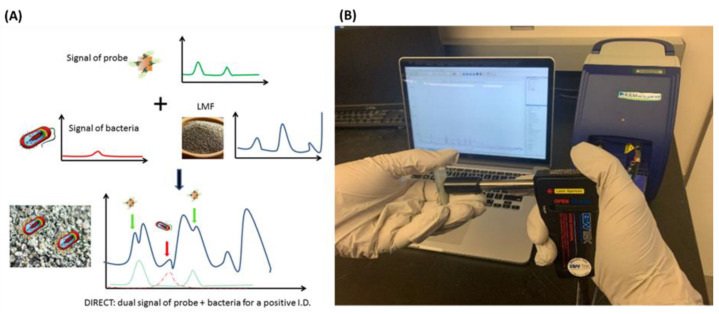
Scheme (**A**) and experiment setup (**B**) of a dual immunological Raman-enabled crosschecking test for detection of contamination in LMF. Reprinted with permission [223], copyright 2020, MDPI.

In low moisture food (LMF) conditions (water activity less than 0.85), the microbial contamination in food is usually bacteria such as *Salmonella* and *E. coli* [222], which cause foodborne diseases. Recently, a dual immunological Raman-enabled crosschecking test detection of bacteria in LMF was reported (Figure 5) [223]. The limit of detection was 10^2^ CFU/g of bacteria with a detection time of 30–45 min to identify food safety risks in real-time. The detection mechanism is based on molecular recognition of antibody–antigen interaction. The dual immunological Raman platform was performed as a model food system of black pepper powder and egg powder.

#### 4.2.2. Nanotechnology Incorporated in Molecular Assays

A molecular assay is a collection of techniques, which utilizes the principle of specific DNA/RNA sequence amplification to identify individual pathogens, including biological contaminants in foods. Generally, a molecular assay contains three main steps: DNA/RNA extraction, DNA/RNA amplification, and detection [224,225,226]. Nanotechnology can be incorporated in molecular assays through these three steps: extraction, amplification, and detection.

For the extraction of DNA/RNA from foodborne pathogens, nanomaterials proved their ability for on-site DNA/RNA purification. A rapid method for multiplex detection of *Salmonella enteritidis* (Gram-negative bacteria) and *L. monocytogenes* (Gram-positive bacteria) was performed in raw milk by using amino-modified silica-coated magnetic nanoparticles for DNA extraction and polymerase chain reaction for DNA amplification [227]. This method was successfully used for multiplex detection of *S. enteritidis* and *L. monocytogenes* with the limits of detection of 15 and 25 CFU/mL, respectively. Yang et al. developed nanoparticle-based immunomagnetic separation coupled with real-time polymerase chain reactions (PCR) for rapid detection of *L. monocytogenes* in milk [228]. In this study, *L. monocytogenes* were separated by immunomagnetic separation using magnetic nanoparticles. Then, the separated *L. monocytogenes* were subjected to DNA extraction and real-time PCR. *L. monocytogenes* were detected in milk samples with a concentration as low as 10^2^ CFU/0.5 mL.

For the amplification, Cui et al. reported that single-walled carbon nanotubes can increase the polymerase chain reaction (PCR) efficiency at a concentration range of less than 3 µg/µL [229]. Zhang et al. also reported the beneficial effect of single-walled carbon nanotubes and multi-walled carbon nanotubes to enhance the PCR efficiency [230]. The hybrid PCR chip comprised of anodic aluminum oxide with internalization of AuNPs significantly enhanced the PCR efficiency and SERS directly detection of *E. coli* as early as the 10th thermal cycle [231]. Li et al. reported a nanoparticle PCR method with the assistance of AuNPs to increase the specificity of PCR [232]. Similarly, the enhancement of PCR efficiency by using AuNP was also demonstrated by Li et al. [233].

For monitoring DNA amplification, Teixeira et al. reported multifunctional AuNP for SERS-based detection of *L. monocytogenes* combined with loop-mediated isothermal amplification (LAMP) [234]. The developed multifunctional AuNPs assisted LAMP in three ways: stabilizing agent, Raman reporter, and chelating agent of magnesium (II) ions. This LAMP coupled with SERS through the multifunctional AuNPs method showed a significantly higher sensitivity than the LAMP-turbidity detection (102 pg/µL vs 1 ng/µL of target DNA). Garrido-Maestu et al. reported a combination of LAMP with AuNPs for rapid detection of *Salmonella* spp. in chicken, turkey, and egg products [235]. This AuNPs-LAMP technique achieved a very low limit of detection (10 CFU/25 g) and the results can be observed by the naked eye.

### 4.3. Detection of Micro/Nanoplastics (MP/NPs)

In recent years, with the extensive use of plastics, micro/nanoplastics (MP/NPs)-related contamination gained the attention of the general public due to the potential threat posed by their presence in several aspects of the environment and human health [236,237,238]. Particularly, MNPs can enter the animal or human body via the ingestion of contaminated foods or packaged beverages [239]. There is an urgent need for a new approach for quantifying MP/NPs with great accuracy, simplicity, and rapid detection [240,241]. Currently, the promising approaches for MP/NPs in food are mainly vibrational spectroscopy (FITR, Raman) and electrochemical analysis toward nanostructure design and development.

**Figure 6:**
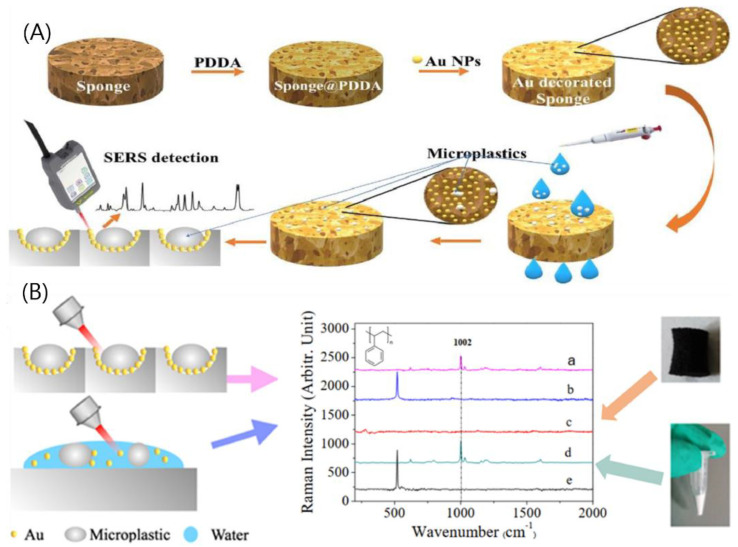
(**A**) A schematic of SERS detection of MNPs. (**B**) Measure the Raman spectra of polystyrene in the sponge-supported Au NPs and polystyrene miss Au colloid. Reprinted with permission [245], copyright 2021, Elsevier. Measure the Raman spectra of polystyrene in SA (a), polystyrene mis Au colloid (b), SA black (c), polystyrene solid (d) and Au solid (e) respectively.

With the use of Raman spectroscopy, several studies were adapted for MNP detection in food samples, using the combination of nanostructure-SERS [242]. For example, an online Raman spectroscopic was applied for polyethylene MNP detection using perfluorocarbon as a particle-capturing medium [243]. In other works, the silver colloid-assisted SERS was introduced for MNP detection in water [244]. As a result, silver colloid helped for enhancing the SERS signal, which appeared in more than two peaks of MP/NPs. This method could effectively identify different types of plastic particles. Yin et al. introduced a sensitive detection method based on SERS for trace microplastics in non-pretreated water samples [245]. In this study, gold nanoparticle-modified sponge substrates could effectively capture and concentrate microplastics, hence controlling the SERS signal intensity, as shown in Figure 6. In conclusion, although this approach still needs more research and development for on-site MP/NPs detection, it greatly displayed more possibility for the rapid detection of MNPs in food samples in the future.

The electrochemical sensors for MNPs were widely applied to detect MP/NPs and extended to the infield testing of different types of samples without prior purification or isolation. Typical electrochemical behavior sensing of MNPs is based on label-free electrochemical impedance spectroscopy (EIS), amperometry, and voltammetry [246]. In fact, the plasticizers may migrate out of plastics into foods, which are then ingested and cause toxicological effects, such as endocrine disruption, carcinogenicity, and bioaccumulation potential [247]. The ultrasensitive electrochemical detection of dibutyl phthalate (DBP), a common plasticizer, was developed by a phthalic acid group-specific aptamer modified on AuNPs that functionalized graphene oxide nanoplatelets and ionic liquid nanocomposite. The sensor exhibits a limit of detection of ≤0.042 pg/mL [248]. The presence of bisphenol-A in foods was reported to cause endocrine disruption, which imitates human hormones and interferes with other biological products in the body [249,250,251]. Different types of nanostructure were developed for electrochemical sensing of bisphenol-A, such as graphene oxide and β-Cyclodextrin-functionalized multi-walled carbon nanotubes (MWCN) with a limit of detection of 6 nM [252], and nanocomposite of MWCN with copper ferrite with a limit of detection of 3.2 nM [253]. A dual-mode competitive immunosensor made of PEI functionalized nitrogen-doped graphene-CoSe_2_/gold nanowire was designed for the detection of DBP. A tunable 3D-printed microfluidic resistive pulse sensor, based on silver wire, which was fabricated by lithograph, could monitor the microplastics in tea bags [254].

## 5. Other Nanomaterial-Based Methodology

### 5.1. Surface Plasmon Resonance

Surface plasmon resonance (SPR) can be defined as charge-density oscillation at the interface when light passes through a substrate and is reflected by the metal-dielectric interface [255]. Sensor-based SPR is a label-free detection method that provides a reliable platform for the highly specific detection of various analytes, such as pollutants, antibiotics, biomolecules, pesticides, insecticides, herbicides, microorganisms, and microbial toxins [256,257]. Nanomaterials were utilized to employ smart layers of the SPR system, which orient the immobilization of the bio-receptors. The principle behavior of SPR-based sensors typically includes fiber-optic SPR [258,259], SPR imaging [260,261], and localized SPR [262,263]. Recently, the four SPR biosensors consisting of prism, Ag, graphene, affinity layer, and sensing medium, were studied to detect bacteria in drinking water [264]. The as-designed SPR sensor exhibited a high sensitivity of *Escherichia coli* (223.63°/RIU) and *Vibrio cholera* (199.87°/RIU). Pesavento and coworkers described a platform of SPR-optical fiber-molecularly imprinted polymer for the detection of furfural in wine [258]. The limit of detection of furfural (2-furaldeheide) was 0.004 mg/L.

### 5.2. Nanoenzymes and Nanopore Sensing

Different nanoenzyme and nanopore techniques were described as powerful analytical tools for food contaminant detection [265,266]. The concept of enzyme-mimetic nanomaterials is diverse in types of analytes. Thus, different nanostructures were fabricated as artificial nanoenzyme mimics. The sensor behavior of nanoenzyme-based detection performs in various analytical methods, such as fluorescence, colorimetric and electrochemical assay, SERS, and electrochemiluminescence [265]. In food contaminant detection, nanoenzyme-based sensors were used for the determination of various endogenous factors and exogenous contaminants in foods at very low concentrations. For instance, the aptasensor made of CdTe/Cds/ZnS quantum dot and modified gold nanorod was prepared for Aflatoxin B1, one of the common mycotoxins, detection with a limit detection of 0.12 pM [267]. The main types of nanomaterials for nanoenzyme techniques included are metal [268,269], metal oxide- [270,271], and carbon-based nanoenzymes [272].

Platforms consisting of nanopore sensing for analytes of biological agents, such as drugs, proteins, and pathogens, is an attractive topic these days [273]. The principle of nanopore sensors is the attaching of molecular recognition analytes that could bind specifically to the synthetic or biological nanopores. To date, nanopore sequencing is considered one of the most advanced techniques for the detection of foodborne microorganisms belonging to this sensing concept due to its high sensitivity, real-time, and low turnaround time [266]. The most recent nanopore sequencing technology, was improved and developed by Oxford Nanopore Technologies [274,275,276]. By embedding a nanohole in a thin membrane and recording the electrochemical signal, nanopore technology can investigate nucleic acids and other biomacromoleculars, which allows the identification of pathogens in food, such as bacteria, viruses, or toxins [274]. Metagenomic analysis-based nanopore sequencing allows the possibility of detecting multiple identifications of viable bacteria [277,278]. Nanopore-based aptasensor was described in the determination of vanillin, a popular favorite additive in food, with a limit of detection of 500 pM. The principle of sensing is based on the high-selective aptamer Van_74 with a high binding affinity of vanillin [279].

### 5.3. Reticular Materials-Based Sensor

Reticular chemistry is applied in many fields due to its unique structure and properties based on two cutting-edge porous framework materials comprised of both organic and inorganic components [280,281]. Metal-organic frameworks (MOFs) and covalent-organic frameworks (COFs) perform the characteristics of flexibility in composition, structure, and pores that are associated with the potential for the development of biosensor platforms. The integration of the structure and functions of MOFs and COFs enriches their structures and properties and shows the great potential application in sensing [282]. Generally, the design of MOFs for sensing applications includes MOFs-based SERS [283,284], MOFs-based electrochemical [285,286], and MOFs-based biosensing [287,288,289]. Bhardwaj and coworkers described a bioconjugate of antibody and MOF for highly sensitive optical sensing of *S. aureus* [290]. The amine-functionalized MOF NH_2_-MIL-53(Fe), with an antibody to form a bio probe, has the potential in the detection of *S. aureus* with a limit of detection of 85 CFU/mL, and is feasible for further detection in cream pastry samples. Integration of numerous MOFs and MOFs/COFs was synthesized for the detection of toxins. The electrochemical aptasensor was made of FeMOF-based composite for dual-enzyme-driven target recycling for the detection of patulin, a toxic chemical contaminant, in apple juice [291]. The limit of detection reached 0.217 fg/mL, reproducibility, and high precision with an RDS of 2.69–4.98%. In other work, manganese-based MOF was employed in the electrochemical sensor for highly sensitive cadmium ion detection in water with a limit of detection of 0.12 ppb [292]. Liu and a coworker reported a new cluster of MOF (Me_2_NH_2_)Cd_3_(OH) and triazine backbones for selective luminescent detection of Hg^2+^ [293]. The hybrid of MOF and COF also exhibits great potential in sensing due to its unique properties of low background noise, high signal-to-noise ratio, and rapid response [294]. The nanostructure of Ce-MOF@COF hybrid was constructed [295]. In this work, the sensing behavior was due to label-free sensitive electrochemical aptasensor for the detection of oxytetracycline in aqueous samples, including water and milk. The Ce-MOF@COF showed benefits in its crystal and chemical structure, large specific surface area, and interpenetrated morphologies.

### 5.4. Photothermal Assays

A large number of nanomaterials was developed to apply in the relative photothermal approach of biological detection. Li and coworkers reported photothermal soft nanoballs made of Cu_2−x_Se nanocrystals and liposomes for immunoassay detection of mycotoxin aflatoxin B1 [296]. The as-prepared photothermal nanostructure works as a thermometer allowing the plasmonic photothermal light-to-heat conversion via photon-electron-phonon coupling. In other work, the MoO_3−x_ nanoparticle was used as a substoichiometric photothermal conversion for the quantitative determination of *E. coli* O157:H7 [297]. The MoO_3−x_ nanoparticle exhibited an excellent photothermal conversion with an efficiency of 42.9% under an 808 nm laser. A bifunctional colorimetric immunosensor and photothermal effect was designed by using peroxidase mimetic and nickel oxide nanoparticles to detect and kill Salmonella typhimurium in milk. The limit of detection of pathogen concentrations was 10 CFU/mL [298]. Gold nanoparticle also has a photothermal effect [299,300]. The incorporation of AuNPs with an immuno-filtration strip is a powerful tool for the routine monitoring of foodborne pathogen bacteria. The thermal contrasts caused by the photothermal effect were proportional to bacteria concentrations. The limit of detection of the designed sensor was 1.95 × 10^4^ CFU/mL for *E. coli* O157:H7 detection [300]. The photothermal sensor-based AuNPs was successfully developed as a portable test strip with excellent detectability. The photothermal effect produced by AuNPs was captured on the test line, and the signal could be recorded by the reader and could be used for the quantitative detection of residues of food hazards.

## 6. Conclusions

This review was organized to describe recent nanotechnologies for food safety analysis on the basis of different types of food contaminants. Food contaminants generally fall into two main categories: chemical contaminants and biological contaminants. Chemical contaminants in foods involve heavy metals (lead, mercury, copper, cadmium, arsenic, etc.) and antibiotic residues (kanamycin, chloramphenicol, 2-mercapto-5-methyl-1,3,4-thiadiazole, etc.). For the detection of chemical contaminants, nanotechnology can incorporate colorimetric analysis for the naked-eye readout of results and serves as an on-site detection method. As another strategy for the detection of chemical contaminants in foods, SERS-based methods along with significant futures of nanomaterials provide ultrasensitive target analysis. Nanotechnology can also be incorporated in electrochemical assays in order to improve the analytical performance for food analysis by electrode modifications and signal tags. For detection of biological contaminants in foods, nanotechnology can be incorporated in both immunoassays and molecular assays to enhance specificity, sensitivity, and accuracy. Additionally, other nanomaterial-based methodologies, such as surface plasmon resonance, nanoenzymes, and nanopore sensing, reticular materials-based sensors, and photothermal assays are efficient for food monitoring.

## Figures and Tables

**Table 1:** The reference values of heavy metals.

Heavy Metals	Reference Value	Agency	Reference
Methylmercury	0.3 μg/kg/day	ATSDR, 1999	[50]
Chromium (III)	300 μg/kg/day	EFSA, 2014	[51]
Chromium (VI)	3 μg/kg/day	EPA, 1998	[52]
Lead	0.16 μg/kg/day	FDA, 2018	[53]
Cadmium	1 μg/kg/day	EPA, 1989	[54]
Nikel	2.8 μg/kg/day	EFSA, 2025	[55]
Strontium	130 μg/kg/day	WHO, 2010	[56]
Zinc	0.43 μg/kg/day	SCF, 2003	[56]
Iron	0.8 μg/kg/day	WHO/FAO, 2010	[56]
Palladium	0.5 μg/kg/day	WHO/FAO, 2010	[56]
Inorganic Arsenic	0.3 μg/kg/day	ATSDR, 2007	[50]
